# Genetic Characterization of Foot-and-Mouth Disease Viruses, Ethiopia, 1981–2007

**DOI:** 10.3201/eid1509.090091

**Published:** 2009-09

**Authors:** Gelagay Ayelet, Mana Mahapatra, Esayas Gelaye, Berhe G. Egziabher, Tesfaye Rufeal, Mesfin Sahle, Nigel P. Ferris, Jemma Wadsworth, Geoffrey H. Hutchings, Nick J. Knowles

**Affiliations:** National Veterinary Institute, Debre Zeit, Ethiopia (G. Ayelet, E. Gelaye, B.G. Egziabher); Institute for Animal Health, Woking, UK (G. Ayelet, M. Mahapatra, N.P. Ferris, J. Wadsworth, G.H. Hutchings, N.J. Knowles); National Animal Health Diagnosis and Investigation Centre, Sebeta, Ethiopia (T. Rufeal, M. Sahle)

**Keywords:** FMD virus, serotype, phylogenetic tree, vaccine strain, Ethiopia, viruses, research

## Abstract

Foot-and-mouth disease (FMD) is endemic to sub-Saharan Africa. To further understand its complex epidemiology, which involves multiple virus serotypes and host species, we characterized the viruses recovered from FMD outbreaks in Ethiopia during 1981–2007. We detected 5 of the 7 FMDV serotypes (O, A, C, Southern African Territories [SAT] 1, and SAT 2). Serotype O predominated, followed by serotype A; type C was not recognized after 1983. Phylogenetic analysis of virus protein 1 sequences indicated emergence of a new topotype within serotype O, East Africa 4. In 2007, serotype SAT 1 was detected in Ethiopia and formed a new distinct topotype (IX), and serotype SAT 2 reappeared after an apparent gap of 16 years. The diversity of viruses highlights the role of this region as a reservoir for FMD virus, and their continuing emergence in Ethiopia will greatly affect spread and consequent control strategy of the disease on this continent.

## Abstract 2

Virus diversity indicates a virus reservoir in this country.

Foot-and-mouth disease virus (FMDV) is the causative agent of a highly contagious and economically devastating disease of cloven-hooved domestic and wild animals. It can cause a high number of deaths among young animals and production losses in adults and is the single most limiting constraint to international trade of live animals and animal products. FMDV belongs to the genus *Aphthovirus* in the family *Picornaviridae* and possesses a single strand of positive-sense RNA genome. It has a high mutation rate because the viral RNA–dependent RNA polymerase lacks proofreading ability, resulting in 7 immunogenically distinct serotypes (O, A, C, Southern African Territories [SAT] 1, SAT 2, SAT 3, and Asia 1) and numerous and constantly evolving variants showing a spectrum of antigenic diversity. Vaccination is an effective way to control FMD; however, the protection conferred by vaccination or infection is usually serotype specific and sometimes incomplete within a serotype (*1**–**3*).

FMD is endemic to sub-Saharan Africa; widespread outbreaks of clinical disease occur during most years (*4*,*5*). Of the 7 serotypes (except Asia 1), 6 have reportedly occurred on the continent, and disease control becomes more complicated because of marked regional differences in the distribution and prevalence of various serotypes and intratypic variants (*4**–**6*). FMD was first recorded in Ethiopia in 1957 when serotypes O and C were detected (*7*,*8*). Serotypes A and SAT 2 were not identified until 1969 and 1989, respectively (*8*,*9*). During 1988–1991, analysis of outbreak samples from Ethiopia at the National Veterinary Institute (NVI), Debre Zeit, Ethiopia, and at the Food and Agriculture Organization World Reference Laboratory for Foot-and-Mouth Disease (WRLFMD), Institute for Animal Health, Pirbright, UK, identified serotype O and serotype SAT 2 FMDV as the causative agents (*9*). The occurrence of FMD in Ethiopia has apparently increased since 1990; outbreaks throughout the country are reported frequently (*10*). FMD remains largely uncontrolled in the country because vaccination for prophylactic purpose is not being practiced except for a few dairy herds containing exotic animals. With no control and preventive measures in place, FMD causes substantial economic loss to farmers and to the nation from embargoes of livestock and livestock product trade (*11*). To initiate control measures for FMD, the following must be identified: origin of infection, links between outbreaks, extent of genetic variation of the causative viruses, and antigenic relationship of field isolates to the available vaccines.

Phylogenetic analysis of the virus protein (VP) 1 region of FMD viruses has been used extensively to investigate the molecular epidemiology of the disease worldwide. These techniques have helped define genetic relationships between FMDV isolates and geographic distribution of lineages and genotypes; they have also helped establish genetically and geographically linked topotypes and trace the source of outbreaks (*4**,**6**,**12*). Topotypes are defined as geographically clustered viruses that form a single genetic lineage generally sharing >85% (O, A, C, and Asia 1) or >80% (SAT 1, SAT 2, and SAT 3) nucleotide identity in the VP1-coding region.

We report the circulation of 4 of 7 serotypes of FMDV in Ethiopia: serotype O, serotype A, serotype SAT 2, and serotype SAT 1. Emergence of these viruses in Ethiopia will greatly affect spread and consequent control strategy of the disease on this continent because restriction of animal movement between many African countries is limited.

## Materials and Methods

During 1981–2007, epithelial tissues and vesicular fluids were collected from FMD-suspected animals from different areas of Ethiopia and submitted to NVI in Debre Zeit. Bovine samples were collected throughout the country; swine samples were collected only from 1 swine farm (Alagae) in Zeway, Eastern Shoa, during 1986 and 1998; and ovine and caprine samples were collected from Mizan Teferi, Bench Maji, in 2007. No samples came from eastern Ethiopia. The samples were transported from the collection site to the diagnostic laboratory in 0.04 M phosphate buffer (pH 7.2–7.6) with 50% glycerol at 4°C (*13*) and stored at –20°C until tested (*14*). When possible, the same samples, or others collected at the same time as those tested at NVI, were also submitted to the WRLFMD in Pirbright for additional studies.

### Laboratory Diagnosis

Viruses were isolated and serotypes were identified as follows. Established cell layers of either IB-RS-2 (porcine kidney) or BHK-21 (baby hamster kidney) at NVI or primary BTy (bovine thyroid) cells at WRLFMD were inoculated with the suspension of suspected material. Cytopathic effects were noted after 24–48 hours in positive samples. If no cytopathic effect was detected, the cells were passaged at least 1× more before the samples were declared negative. Serotyping of FMDV was carried out by complement fixation test at NVI (*13*) and by antigen-detection ELISA at WRLFMD (*15*).

### Viruses and Primers

A selection of 81 viruses submitted to WRLFMD was further characterized by sequencing of the VP1 gene. The designation and origin of FMDV isolates studied are listed in Technical Appendix Table 1. Three alternative primer combinations were used for the reverse transcription–PCR (RT-PCR) of FMDV serotype O viruses: O-1C244F/EUR-2B52R, O-1C272F/EUR-2B52R, and O-1C283F/EUR-2B52R. Two primer sets were used for each of the other serotypes: serotype A (A-1C562F/EUR-2B52R and A-1C612F/EUR-2B52R), serotype C (C-1C536F/EUR-2B52R and C-1C616F/EUR-2B52R), serotype SAT 1 (SAT1-1C559F/SAT-2B208R and SAT-1U-OS/SAT 2B208R), and serotype SAT 2 (SAT2-P1-1223F/SAT-2B208R and SAT2-1C445F/SAT-2B208R) (Technical Appendix Table 2). Additional internal sequencing primers were used to ensure coverage of the VP1 region on both strands (Technical Appendix Table 2).

### RT-PCR of Virus RNA

RNA extraction and RT-PCR were conducted according to the protocol described previously (*16*), except for the following. The thermal profiles used for amplification of the VP1 sequence of various serotypes were as follows: FMDV O: 42°C for 30 min, 94°C for 5 min, 35 cycles of 94°C for 60 s, 60°C for 60 s, and 72°C for 90 s, followed by a final extension of 72°C for 5 min. Conditions were the same for the other serotypes, except that extension temperatures were 55°C for A and C and 50°C for SAT 1 and SAT 2. After PCR, deoxyribonucleotide triphosphates and primers were removed by using GFX PCR DNA and Gel Band Purification Kit (GE Healthcare, Buckinghamshire, UK) according to the manufacturer’s instructions. The purified PCR product was stored at –20°C until used.

### DNA Sequencing

PCR amplicons were sequenced by using the DTS Quick Start Kit (Beckman Coulter, Fullerton, CA, USA) according to the manufacturer’s instructions and the sequencing primers listed in Technical Appendix Table 2. The sequencing reactions were run on a CEQ8000 Automated Sequencer (Beckman Coulter) according to the manufacturer’s instructions. Sequences determined in this study have been submitted to the EMBL/GenBank/DDBJ databases; accession numbers are shown in Technical Appendix Table 1.

### Phylogenetic Analyses

**Figure 3:**
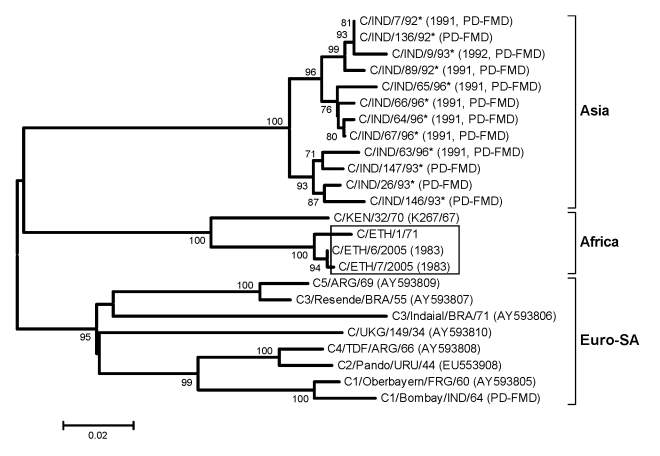
Midpoint-rooted neighbor-joining tree (based on the complete virus protein [VP] 1 coding sequence) showing the relationships between the foot-and-mouth disease virus serotype C isolates from Ethiopia (boxed) and other contemporary and reference viruses. The year in parenthesis indicates the year of sample collection. Scale bar indicates substitutions per site. *Not a reference number assigned by the World Reference Laboratory for Foot-and-Mouth Disease, Pirbright, UK.

Total RNA was extracted from 81 FMD viruses in Ethiopia, and each VP1-coding region was successfully amplified by RT-PCR. The PCR products were directly sequenced on both strands to obtain the complete VP1 sequences, which were compared with the other relevant FMDV VP1 sequences within the same serotype (see Figures 1–5 for database accession numbers).

VP1 nucleotide sequences were aligned by using BioEdit 7.0.5.3 (*17*) and Clustal W (*18*). These alignments were used to construct distance matrices by using the Kimura 2-parameter nucleotide substitution model in the program MEGA 4.0 (*19*). Some previously published sequences of serotype O were incomplete at the 5′ end of the VP1 gene and consisted of 495 nucleotides rather than the full-length 639 nucleotides. Midpoint-rooted neighbor-joining trees were then constructed with MEGA 4.0 software. The robustness of the tree topology was assessed with 1,000 bootstrap replicates by using the model in MEGA 4.0. The serotype C sequences labeled PD-FMD in Figure 3 were supplied by the Project Directorate on FMD, Mukteswar, India (*20*).

### Vaccine Strain Selection

Vaccine strain selection for Ethiopian serotype O isolates was performed at WRLFMD by using the virus neutralization test. Relationship (r_1_) values were determined as described elsewhere (*21*). An r_1_ value of >0.3 was considered a good match with the vaccine strain (*22*).

## Results

### Distribution of FMD

**Figure 6:**
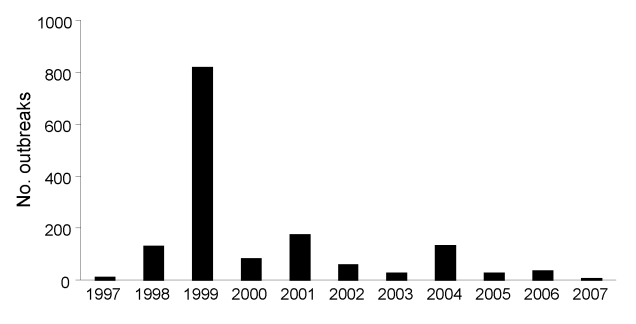
Number of foot-and-mouth disease outbreaks per year in different parts of the country, 1997–2007. Data from Ministry of Agriculture and Rural Development, Ethiopia; data for 1981–1996 not available.

FMD outbreaks occurred every year, but most (821) were reported in 1999 (Figure 6). This finding is consistent with previously reported findings (*10*,*23*) but is probably an underrepresentation of the actual situation. Of the 269 outbreak samples examined, FMDV was isolated from 82.2% (Table 1). During 1981–2007, a total of 5 serotypes (O, A, C, SAT 1, and SAT 2) were identified in bovine, swine, ovine, and caprine samples collected from the outbreak areas. FMDV O was the dominant serotype (73.3%), followed by types A (19.5%), SAT 2 (4.1%), SAT 1 (1.8%), and C (1.3%).

**Table 1:** Species of animals affected and foot-and-mouth disease virus serotypes identified in outbreaks in Ethiopia, 1981–2007*

Species	No. samples tested/no. positive†	Serotype				
		O	A	C	SAT 1	SAT 2
Bovine	250/216	159	43	3	2	9
Swine	7/3	3	–	–	–	–
Sheep	5/1	–	–	–	1	–
Goats	7/1	–	–	–	1	–
Total	269/221	162	43	3	4	9

*SAT, Southern African Territories; –, not identified. †Positive results determined by cytopathic effect.

**Figure 7:**
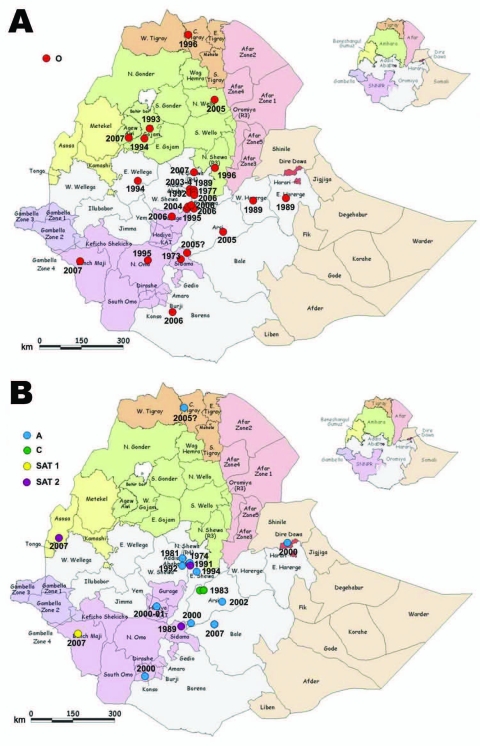
Location of cases of various foot-and-mouth disease (FMD) virus serotypes in the outbreaks of FMD, Ethiopia, 1981–2007, as evidenced by laboratory diagnosis. A) Serotype O, B) serotypes A, C, Southern African Territories (SAT) 1, and SAT 2. All boundaries are approximate and unofficial. Original map produced by United Nations Emergencies Unit for Ethiopia, 2000.

Geographically, the outbreaks were widely distributed. Most were within central Ethiopia, including the Addis Ababa administrative region; the rest were in Ahmara and Tigray in the north, Dire Dawa in the northeast, Beneshangul-Gumuz bordering Sudan in the west, and Southern Nations Nationalities and Peoples Region bordering Kenya and Sudan in the south (Figure 7). In eastern Ethiopia, poor veterinary services and inaccessibility to the area could have resulted in the lack of samples submitted.

In terms of species, cattle were found to be infected with all circulating serotypes of FMDV, whereas swine had only type O (Tables 1 and 2). SAT 2 was recorded in 2007, after an apparent gap of 16 years, from a bovine sample collected from Bambas, Beneshangul-Gumuz, western Ethiopia bordering Sudan (Figure 7, panel B). The first recorded occurrence of FMDV type SAT 1 in Ethiopia was identified from a bovine sample collected in November 2007 from the Mizan Teferi area bordering Kenya (Figure 7, panel B). Analysis of the samples collected from the same region 1 month later, in December 2007, showed involvement of 3 species: cattle, sheep, and goats.

**Table 2:** Serotypes of foot-and-mouth disease viruses isolated in Ethiopia, 1981–2007*

Year	Serotype					Total
	O	A	C	SAT 1	SAT 2	
1981	–	2	–	–	–	2
1982	10	8	–	–	–	18
1983	4	–	3	–	–	7
1984	–	7	–	–	–	7
1985	–	7	–	–	–	7
1986	2	2	–	–	–	4
1987	6	–	–	–	–	6
1988	3	–	–	–	–	3
1989	3	–	–	–	2	5
1990	15	–	–	–	2	17
1991	2	–	–	–	4	6
1992	12	1	–	–	–	13
1993	4	–	–	–	–	4
1994	10	12	–	–	–	22
1995	5	–	–	–	–	5
1996	1	1	–	–	–	2
1997†	–	–	–	–	–	0
1998	10	–	–	–	–	10
1999	16	–	–	–	–	16
2000	3	1	–	–	–	4
2001	12	–	–	–	–	12
2002	–	1	–	–	–	1
2003	3	–	–	–	–	3
2004	26	–	–	–	–	26
2005	4	–	–	–	–	4
2006	7	–	–	–	–	7
2007	4	1		4	1	10
Total	162	43	3	4	9	221

*SAT, Southern African Territories; –, not isolated. †Samples not collected or not received.

### Phylogenetic Analyses

#### Serotype O

**Figure 1:**
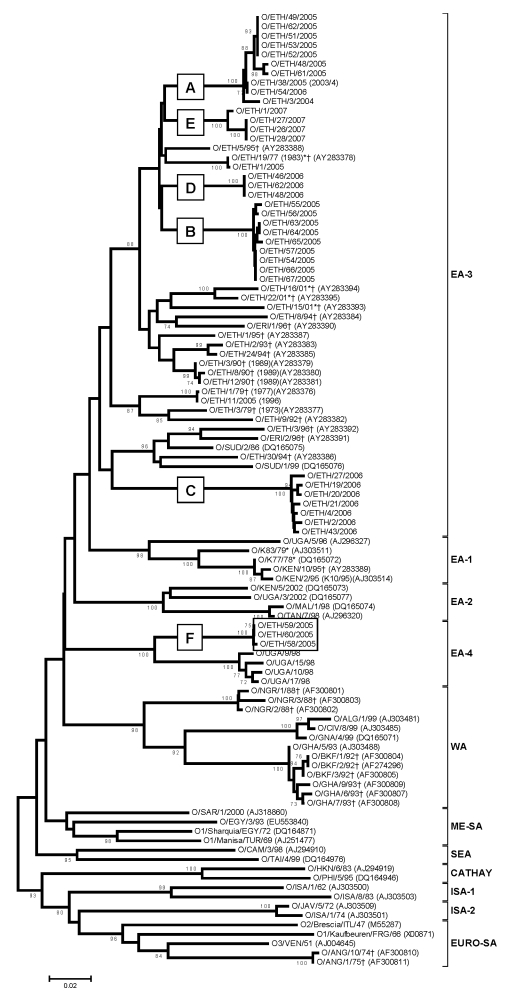
Midpoint-rooted neighbor-joining tree (based on the complete virus protein [VP] 1 coding sequence) showing the relationships between the foot-and-mouth disease virus serotype O isolates from Ethiopia and other contemporary and reference viruses. The 3 isolates from 2005 forming a new topotype East African (EA)-4 are boxed. The year in parenthesis indicates the year of sample collection. Scale bar indicates substitutions per site. *Not a reference number assigned by the World Reference Laboratory for Foot-and-Mouth Disease, Pirbright, UK. †Partial (495-nt) VP1 sequence used.

Of the FMDVs examined, serotype O predominated. All but 3 of the 55 FMDV serotype O Ethiopia isolates examined in our study fell into a single topotype, East Africa (EA)-3 (Figure 1). These 3 samples from 2005 were collected from cattle in the Mizan Teferi area (southwest of Addis Ababa) and formed a new serotype O topotype, which we named EA-4. Four viruses from Uganda in 1998 (≈91% nucleotide identity) also belonged to this topotype. The VP1 sequences of viruses within EA-4 differed by ≈14%–16% from members of the EA-1, EA-2, and EA-3 topotypes. Ethiopia type O viruses isolated during 2003–2007 fell into 6 lineages (A–F; Figure 1), which appeared to be cocirculating in different geographic regions (Figure 7, panel A; Technical Appendix Table 1).

#### Serotype A

**Figure 2:**
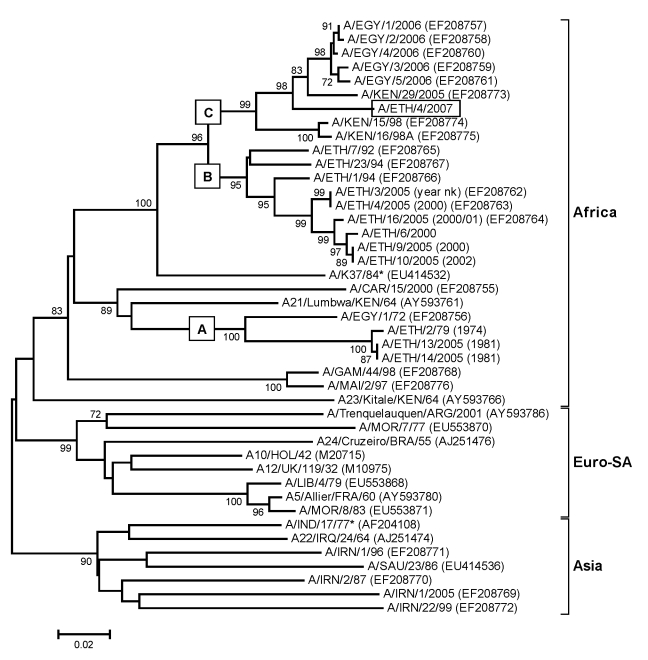
Midpoint-rooted neighbor-joining tree (based on the complete virus protein [VP] 1 coding sequence) showing the relationships between the foot-and-mouth disease virus serotype A isolates from Ethiopia and other contemporary and reference viruses. The isolate from 2007 is boxed. The year in parenthesis indicates the year of sample collection. Scale bar indicates substitutions per site. *Not a reference number assigned by the World Reference Laboratory for Foot-and-Mouth Disease, Pirbright, UK.

All viruses from Ethiopia belonged to the AFRICA topotype (Figure 2) (*6*). Three distinct lineages comprising viruses from Ethiopia were evident; all had high bootstrap support. Lineage A (1979 and 1981) also contained a single virus from Egypt in 1972; lineage B (1992–2002) contained only viruses from Ethiopia; and lineage C (2007) also contained viruses from Kenya (1998 and 2005) and Egypt (2006). Lineage B spanned 11 years and contained viruses isolated from 4 main regions—Oromiya, Tigray, Dire Dawa, and Southern Nations Nationalities and Peoples Region—indicating widespread dispersal of type A viruses (Figure 2). The virus isolated in 2007 (A/ETH/4/2007) was more closely related to the virus isolated from Kenya in 2005 (≈5% nt difference) than to that isolated from Ethiopia in 2000–2002 (≈10% nt difference).

#### Serotype C

Serotype C was not identified after 1983. Phylogenetic analysis showed that all serotype C viruses from Africa belonged to a single lineage (Figure 3), which has been designated the AFRICA topotype (N.J. Knowles, unpub. data). The 2 virus isolates from 1983 were closely related to a virus from Ethiopia in 1971 (≈98.5% nt identity) and grouped with the Kenya vaccine strain, K267/67 (≈94% nt identity) (Figure 3).

#### Serotypes SAT 1 and SAT 2

**Figure 4:**
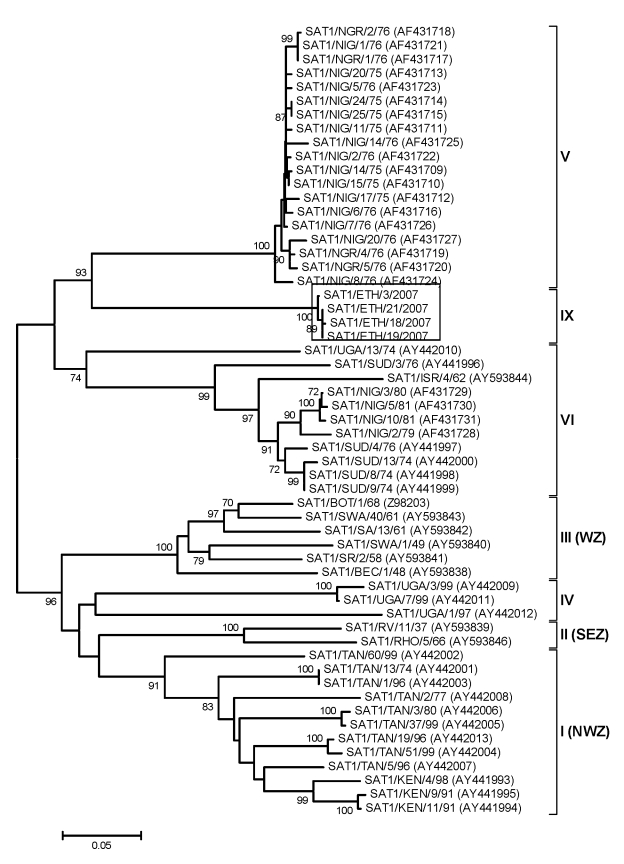
Midpoint-rooted neighbor-joining tree (based on the complete virus protein 1 coding sequence) showing the relationships between the foot-and-mouth disease virus serotype Southern African Territories (SAT) 1 isolates from Ethiopia and other contemporary and reference viruses. The 4 isolates from Ethiopia in 2007 are boxed. The year in parenthesis indicates the year of sample collection. Scale bar indicates substitutions per site. *Not a reference number assigned by the World Reference Laboratory for Foot-and-Mouth Disease, Pirbright, UK.

Genetic characterization of the newly identified SAT 1 isolates from Ethiopia indicates that they are all closely related but distinct from all other SAT 1 viruses from East Africa examined in this study (Figure 4). They were most closely related to viruses from Niger and Nigeria during 1975–1976 (topotype V) (*24*) but were different enough (SAT1/ETH/4/2007 vs. SAT1/NIG/11/75, 23% nt difference) to be classified as a new topotype, which we named topotype IX.

**Figure 5:**
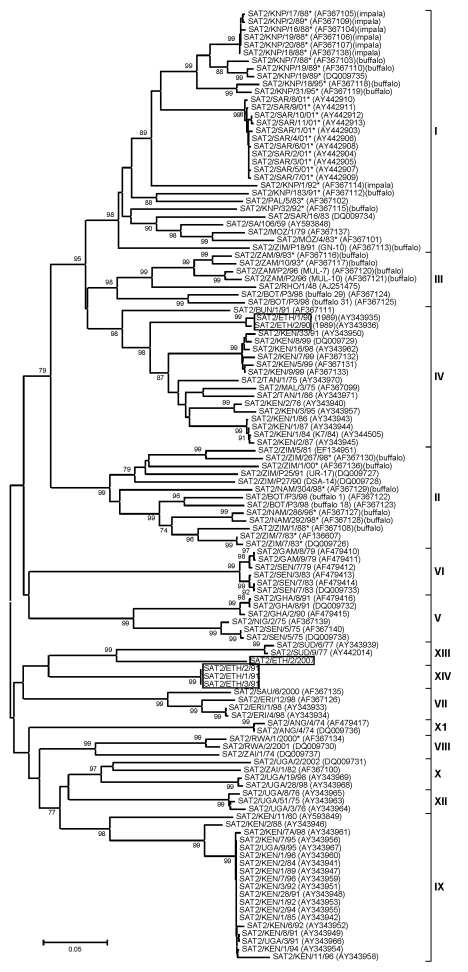
Midpoint-rooted neighbor-joining tree (based on the complete virus protein 1 coding sequence) showing the relationships between the foot-and-mouth disease virus serotype Southern African Territories (SAT) 2 isolates from Ethiopia and other contemporary and reference viruses. The SAT 2 isolates from Ethiopia under lineage IV, XIII, and IVX are boxed. The year in parenthesis indicates the year of sample collection. Scale bar indicates substitutions per site. *Not a reference number assigned by the World Reference Laboratory for Foot-and-Mouth Disease, Pirbright, UK.

The first isolation of SAT 2 was in 1989 from a sample collected from cattle raised on Leben Ranch, Borena Zone, in southern Ethiopia (*9*); the virus was detected for the next 2 years but not again until 2007, an apparent gap of 16 years. Phylogenetic analysis of SAT 2 viruses from Ethiopia shows 3 distinct topotypes: IV (isolates from 1989), XIV (isolates from 1991), and XIII (single isolate from 2007) (Figure 5). Topotype IV has been detected in other African countries (Burundi, Malawi, Kenya, and Tanzania); topotype XIV was isolated only from Ethiopia. The new 2007 SAT 2 isolate from Beneshangul-Gumuz, Ethiopia (regional state bordering Sudan), did not group under either of the above-mentioned topotypes; rather, it could be assigned to topotype XIII along with Sudan isolates (SUD/6/77 and SUD/9/77, ≈81% nt identity) supported by a bootstrap value of 99% (Figure 5).

### Vaccine Strain Selection

For vaccine strain selection for the new topotype of serotype O FMDV (EA-4), serologic tests were conducted. The extent of in vitro cross-neutralization of O/ETH/58/2005 by antiserum against serotype O vaccine strains was evaluated. The match against vaccine strains O_1_ Manisa and O_1_ Lausanne were above the cutoff value of 0.3 (r_1_ 0.42 and 0.32, respectively), which indicated that both strains can confer protection for the new topotype (*22*). Similarly, representative samples from topotype EA-3 (O/ETH/9/2005 and O/ETH/10/2005) showed the O_1_ Manisa vaccine strain to be the vaccine of choice (r_1_ 0.63 and 0.52, respectively).

## Discussion

Similar to our findings, previous studies also have indicated serotype O to be highly prevalent and a dominant serotype causing most of the outbreaks in Ethiopia (*7*,*8*,*23*). The molecular epidemiology of serotype O has been well studied (*6*,*12*). Our study showed the existence of a fourth FMDV serotype O EA topotype. On the basis of comparison of sequence data of the VP1 gene, existence of 8 serotype O topotypes has been demonstrated within samples collected around the world (*12*). Among those, 2 topotypes were found in Africa, 1 in East Africa, and 1 in West Africa. Sangare et al. (*25*) described 7 genotypes of serotype O virus, 4 of which contained isolates from Africa. Samuel and Knowles (*12*) reported that isolates from Kenya and Uganda formed part of a single East African topotype (EA); viruses from Ethiopia, Tanzania, and Eritrea belonged to the Middle East–South Asia (ME-SA) topotype. However, their study was based on partial VP1 sequence data (3′ end of the gene), and it has been suggested that the relationships observed may have resulted from previous recombination events (*26*). Knowles et al. (*26*) renamed the EA topotype as EA-1 and identified 2 additional EA topotypes: EA-2 in Burundi, Kenya, Malawi, Rwanda, Tanzania, Uganda, and northern Zambia and EA-3 in Eritrea, Ethiopia, and Sudan.

Since 1983, serotype C seems to have disappeared from Ethiopia (Figure 3). However, a recent report of serotype C–specific antibodies in cattle in Ethiopia (*27*) indicates that circulation of serotype C viruses in Ethiopia may have gone unnoticed. No outbreaks of serotype C have been reported in Europe since 1989 (Italy), in South America since 2004 (Brazil), in Asia since 1995 (India and the Philippines) or 1996 (Nepal), and in Africa since 2004 (Kenya) (*28*). Therefore, investigations of the epidemiology of serotype C viruses in Ethiopia are urgently needed.

Genetic characterization of SAT type viruses is well documented for the southern African region; emphasis has been on viruses isolated from the African buffalo (*Syncerus caffer*) (*29*–*32*) and on cattle viruses from West Africa (*33*) and East Africa (*24*). On the basis of nucleotide sequence analysis of a portion of the viral genomes obtained from buffalo and domestic animals in sub-Saharan Africa, 14 independently evolving viral genotypes were identified for SAT 2, 8 for SAT 1, and 6 for SAT 3 (*5*). FMDV SAT 1 was first isolated in Ethiopia (Bench Maji, Southern Nations Nationalities and Peoples Region) in 2007 from samples collected from 3 species: cattle, sheep, and goats. Although SAT 1 has not been previously reported in Ethiopia (*7*,*8*,*24*), it might be circulating within wildlife and infrequently transmitted to domestic animals. SAT 2 may have been recently introduced by animal movement across the border with Sudan because SAT 2 is endemic to Sudan (*5*,*34*). Recent presence of serotype SAT 2–specific antibodies in cattle in Ethiopia has been reported (*27*). Two explanations are possible: 1) the virus is present in Ethiopia but has not been detected because all outbreaks are not reported or investigated, or 2) type SAT 2 viruses circulate subclinically in Ethiopia, possibly in wildlife.

FMD is endemic to Ethiopia as it is in all the bordering countries—Eritrea in the northeast, Sudan in the west, Kenya in the south, and Somalia in the east—and restriction of animal movement is limited. A large number of wildlife, including African buffalo (particularly in the Mago and Omo national parks), could act as FMDV reservoirs. The association of SAT serotypes with wildlife, particularly African buffalo, has been indicated (*5*,*24**,**35*,*36*). Individual buffalo can harbor the virus for as long as 5 years, and an isolated buffalo herd can maintain FMDV for 24 years (*37*). Transmission of virus from infected buffalo to other susceptible animals in close contact has been demonstrated (*38*–*40*). Therefore, transmission of FMDV by cattle movement or from wild animals to domestic animals is likely and may play a role in FMD outbreaks and in the appearance of new topotypes in Ethiopia.

The veterinary infrastructure for FMD disease surveillance and also the outbreak reporting system, which have not been efficient because of things such as financial constraints and difficulty accessing some regions, has improved considerably since the 1990s. Hence, this study may not be a true reflection of the number of serotypes/topotypes present in Ethiopia because not all outbreaks are reported or investigated due to the endemic nature of the disease. Therefore, comprehensive studies, including wildlife for molecular epidemiology and representative samples from all regions, are needed.

In terms of selecting vaccine strains, assessing the threat of SAT 1 and SAT 2 viruses in Ethiopia is difficult because SAT viruses often appear sporadically and then disappear in Ethiopia (only 1 SAT 1 and 4 SAT 2 samples have been isolated in 2007, and to date, no SAT outbreak has been reported in Ethiopia). Therefore, regular monitoring of the circulation of these viruses in livestock may help with selection of appropriate vaccine strains for FMD control.

In conclusion, the epidemiology of FMD in Ethiopia is complex because multiple serotypes of the virus (O, A, SAT 1, and SAT 2) circulate, 4 host species (cattle, sheep, goats, and pig) are involved, and high numbers of wildlife (especially African buffalo) cross the borders of neighboring countries uncontrolled. In addition, lack of prophylactic vaccination and veterinary infrastructure to handle outbreaks on a large scale greatly contribute to the frequent occurrence of the disease and make control of FMD extremely challenging. Regular monitoring and more detailed investigation are needed to formulate an efficient vaccine-based FMD control strategy for Ethiopia.

## Supplementary Material

Technical Appendix Table 1-2 Genetic Characterization of Foot-and-Mouth Disease Viruses, Ethiopia, 1981-2007
