# Supplementary material for: Genetic Characterization of Foot-and-Mouth Disease Viruses, Ethiopia, 1981–2007
Source: Emerg Infect Dis. 2009 Sep;15(9):1409–17. doi: 10.3201/eid1509.090091 (PMC2819860; doi:10.3201/eid1509.090091)
Supplement: Technical Appendix Table 1-2 — Genetic Characterization of Foot-and-Mouth Disease Viruses, Ethiopia, 1981-2007 [file 09-0091_Techapp-s1.pdf]

Genetic Characterization of Foot-and-Mouth Disease Viruses, Ethiopia, 1981–2007

Technical Appendix

Technical Appendix Table 1. Designations and origins of foot-and-mouth disease viruses used in phylogenetic analyses of viruses from Ethiopia collected during 1981–2007

Serotype	Topotype	Sublineage	WRLFMD no.	Date collected	Place (District, Zone, Region)	Species	Accession no.
O	EA-3	ND	ETH/1/79	1977 Oct 8	Debre Zeit, East Shoa, Oromiya	Cattle	AY283376
O	EA-3	ND	ETH/3/79	1973 Mar 31	Neguele, Sidamo, Oromiya	Cattle	AY283377
O	EA-3	ND	ETH/19/77†	1983	NK	NK	AY283378
O	EA-3	ND	ETH/1/2005	NK	Awassa, Sidama, SNNPR	Cattle	FJ798106
O	EA-3	ND	ETH/3/90	1989 Aug 24	Nefas Silk Lafto, Addis Ababa	Cattle	AY283379
O	EA-3	ND	ETH/8/90	1989 Dec 1	West Hararge, Oromiya	Cattle	AY283380
O	EA-3	ND	ETH/12/90	1989 Dec 1	East Hararge, Oromiya	NK	AY283381
O	EA-3	ND	ETH/9/92	1992 Dec 2	Kotebe, Addis Ababa	Cattle	AY283382
O	EA-3	ND	ETH/2/93	1993 Aug 16	Shenge, West Gojam, Amhara	NK	AY283383
O	EA-3	ND	ETH/8/94	1994 Feb 2	Highland area of Eastern Ethiopia	Cattle	AY283384
O	EA-3	ND	ETH/24/94	1994 May 3	Markuma, West Gojam, Amhara	NK	AY283385
O	EA-3	ND	ETH/30/94	1994 Aug 15	Seyo, Wellega, Oromiya	NK	AY283386
O	EA-3	ND	ETH/1/95	1995 Oct 2	Melge, Wondo Genet, Shashemene, SNNPR	Cattle	AY283387
O	EA-3	ND	ETH/5/95	1995 Dec 7	Boditi, North Omo, SNNPR	Cattle	AY283388
O	EA-3	ND	ETH/3/96	1996 Mar 23	Tigray	Cattle	AY283392
O	EA-3	ND	ETH/11/2005	1996 Mar 6	Debre Birhan, North Shoa, Amhara	Cattle	FJ798107
O	EA-3	ND	ETH/15/01†	2001	NK	NK	AY283393
O	EA-3	ND	ETH/16/01†	2001	NK	NK	AY283394
O	EA-3	ND	ETH/22/01†	2001	NK	NK	AY283395
O	EA-3	A	ETH/38/2005	2003–04	Addis Ababa	Cattle	FJ798108
O	EA-3	A	ETH/3/2004	2004 Jan 1	Shashemene, East Shoa, Oromiya	Cattle	FJ798109
O	EA-3	A	ETH/48/2005	2005 Jan 9	Guba Lafto, North Wollo, Amhara	Cattle	FJ798110
O	EA-3	A	ETH/49/2005	2005 Jan 9	Guba Lafto, North Wollo, Amhara	Cattle	FJ798111
O	EA-3	A	ETH/51/2005	2005 Jan 9	Guba Lafto, North Wollo, Amhara	Cattle	FJ798112
O	EA-3	A	ETH/52/2005	2005 Jan 9	Guba Lafto, North Wollo, Amhara	Cattle	FJ798113
O	EA-3	A	ETH/53/2005	2005 Jan 9	Guba Lafto, North Wollo, Amhara	Cattle	FJ798114
O	EA-3	A	ETH/61/2005	2005 Jan 9	Guba Lafto, North Wollo, Amhara	Cattle	FJ798115
O	EA-3	A	ETH/62/2005	2005 Jan 9	Guba Lafto, North Wollo, Amhara	Cattle	FJ798116
O	EA-3	A	ETH/54/2006	2006 Jan 1	Kality, Oromiya	Cattle	FJ798117
O	EA-3	B	ETH/54/2005	2005 Feb 15	Robe, Arsi, Oromiya	Cattle	FJ798118
O	EA-3	B	ETH/55/2005	2005 Feb 15	Robe, Arsi, Oromiya	Cattle	FJ798119
O	EA-3	B	ETH/56/2005	2005 Feb 15	Robe, Arsi, Oromiya	Cattle	FJ798120
O	EA-3	B	ETH/57/2005	2005 Feb 15	Robe, Arsi, Oromiya	Cattle	FJ798121
O	EA-3	B	ETH/63/2005	2005 Apr 20	Robe, Arsi, Oromiya	Cattle	FJ798122
O	EA-3	B	ETH/64/2005	2005 Apr 20	Robe, Arsi, Oromiya	Cattle	FJ798123
O	EA-3	B	ETH/65/2005	2005 Apr 20	Robe, Arsi, Oromiya	Cattle	FJ798124
O	EA-3	B	ETH/66/2005	2005 Apr 20	Robe, Arsi, Oromiya	Cattle	FJ798125
O	EA-3	B	ETH/67/2005	2005 Apr 20	Robe, Arsi, Oromiya	Cattle	FJ798126
O	EA-3	C	ETH/2/2006	2006 May 1	Yabello, Boreno, Oromiya	Cattle	FJ798127
O	EA-3	C	ETH/4/2006	2006 May 1	Yabello, Boreno, Oromiya	Cattle	FJ798128
O	EA-3	C	ETH/19/2006	2006 May 1	Yabello, Boreno, Oromiya	Cattle	FJ798129

Serotype	Topotype	Sublineage	WRLFMD no.	Date collected	Place (District, Zone, Region)	Species	Accession no.
O	EA-3	C	ETH/20/2006	2006 May 1	Yabello, Boreno, Oromiya	Cattle	FJ798130
O	EA-3	C	ETH/21/2006	2006 May 1	Yabello, Boreno, Oromiya	Cattle	FJ798131
O	EA-3	C	ETH/27/2006	2006 May 1	Yabello, Boreno, Oromiya	Cattle	FJ798132
O	EA-3	C	ETH/43/2006	2006 Oct 1	Dallocha, Gurage, SNNPR	Cattle	FJ798133
O	EA-3	D	ETH/46/2006	2006 Dec 28	Abernosa, East Shoa, Oromiya	Cattle	FJ798134
O	EA-3	D	ETH/48/2006	2006 Dec 28	Abernosa, East Shoa, Oromiya	Cattle	FJ798135
O	EA-3	D	ETH/62/2006	2006 Dec 1	Ziway, Oromiya	Cattle	FJ798136
O	EA-3	E	ETH/1/2007	2007 Feb 8	Ankesha, Agew Awi, Amhara	Cattle	FJ798137
O	EA-3	E	ETH/26/2007	2007 Dec 1	Fiche, North Shoa, Oromiya	NK	FJ798138
O	EA-3	E	ETH/27/2007	2007 Dec 1	Fiche, North Shoa, Oromiya	Cattle	FJ798139
O	EA-3	E	ETH/28/2007	2007 Dec 1	Fiche, North Shoa, Oromiya	Cattle	FJ798140
O	EA-4	F	ETH/58/2005	2005 Apr 20	Mizan Teferi, Bench Maji, SNNPR	Cattle	FJ798141
O	EA-4	F	ETH/59/2005	2005 Apr 20	Mizan Teferi, Bench Maji, SNNPR	Cattle	FJ798142
O	EA-4	F	ETH/60/2005	2005 Apr 20	Mizan Teferi, Bench Maji, SNNPR	Cattle	FJ798143
A	Africa	A	ETH/2/79	1974 Dec 9	Addis Ababa	Cattle	FJ798144
A	Africa	A	ETH/13/2005	1981	Geferssa, West Shoa, Oromiya	Cattle	FJ798145
A	Africa	A	ETH/14/2005	1981	Geferssa, West Shoa, Oromiya	Cattle	FJ798146
A	Africa	B	ETH/7/92	1992 Oct 3	Alem Gena, West Shoa, Oromiya	Cattle	EF208765
A	Africa	B	ETH/1/94	1994 Feb 2	Highland area of Eastern Ethiopia	Cattle	EF208766
A	Africa	B	ETH/23/94	1994 Mar 9	Nazret, East Shoa, Oromiya	NK	EF208767
A	Africa	B	ETH/6/2000	2000 Jan 1	Konso, SNNPR	Cattle	FJ798147
A	Africa	B	ETH/3/2005	NK	Tigray	Cattle	EF208762
A	Africa	B	ETH/4/2005	2000 Dec 11	Dire Dawa	Cattle	EF208763
A	Africa	B	ETH/9/2005	2000 Dec 27	Coffelle, Arsi, Oromiya	Cattle	FJ798148
A	Africa	B	ETH/16/2005	2000–01	Ambursi, Hadiya, SNNPR	Cattle	EF208764
A	Africa	B	ETH/10/2005	2002 Jan 11	Gobe, Arsi, Oromiya	Cattle	FJ798149
A	Africa	C	ETH/4/2007	2007 Dec 7	Adaba, Bale, Oromiya	Cattle	FJ798150
C	Africa	ND	ETH/1/71	NK	NK	NK	FJ798151
C	Africa	ND	ETH/6/2005	1983 Feb 3	Chilalo, Arsi, Oromiya	Cattle	FJ798152
C	Africa	ND	ETH/7/2005	1983?	Assella, Arsi, Oromiya	Cattle	FJ798153
SAT 1	IX	ND	ETH/3/2007	2007 Nov 10	Mizan Teferi, Bench Maji, SNNPR	Cattle	FJ798154
SAT 1	IX	ND	ETH/18/2007	2007 Dec 6	Mizan Teferi, Bench Maji, SNNPR	Goat	FJ798155
SAT 1	IX	ND	ETH/19/2007	2007 Dec 6	Mizan Teferi, Bench Maji, SNNPR	Sheep	FJ798156
SAT 1	IX	ND	ETH/21/2007	2007 Dec 6	Mizan Teferi, Bench Maji, SNNPR	Cattle	FJ798157
SAT 2	IV	ND	ETH/1/90	1989 Aug 15	Awassa, Sidama, SNNPR	Cattle	AY343935
SAT 2	IV	ND	ETH/2/90	1898 Aug 15	Awassa, Sidama, SNNPR	Cattle	AY343936
SAT 2	XIV	ND	ETH/1/91	1991 May 1	Addis Ababa	Cattle	FJ798158
SAT 2	XIV	ND	ETH/2/91	1991 May 1	Addis Ababa	Cattle	FJ798159
SAT 2	XIV	ND	ETH/3/91	1991 May 1	Addis Ababa	Cattle	FJ798160
SAT 2	XIII	ND	ETH/2/2007	2007 Sep 7	Bambas, Asosa, Beneshangul-Gumuz	Cattle	FJ798161

*ND, not defined; WRLFMD, Food and Agriculture Organization World Reference Laboratory for Foot-and-Mouth Disease; NK, not known; SNNPR, Southern Nations, Nationalities, and Peoples Region; SAT, Southern African Territories.
†Not a WRLFMD reference number.

Technical Appendix Table 2. Location and sequences of oligonucleotide primers used for reverse transcription–PCR and sequencing

Oligonucleotide	Sequence (5’→3’)	Sense	Location	Serotype	Method used
NK72	GAAGGGCCCAGGGTTGGACTC	–	2A/2B	All	Sequencing
EUR-2B52R	GACATGTCCTCCTGCATCTGGTTGAT	–	2B	O, A, C	RT-PCR
O-1C272F	TBGCRGGNCTYGCCCAGTACTAC	+	VP3	O	RT-PCR and sequencing
O-1C244F	GCAGCAAAACACATGTCAAACACCTT	+	VP3	O	RT-PCR
O-1C283F	GCCCAGTACTACACACAGTACAG	+	VP3	O	RT-PCR
O-CRH2F	GAYTACGCSTACACSGCGTC	+	VP3	O	Sequencing
O-1D293F	TGGAYAACACCACYAAYCCAAC	+	VP1	O	Sequencing
O-1D296F	ACAACACCACCAACCCAAC	+	VP1	O	Sequencing
O-1D296bF	ACAACACCACCAATCCAAC	+	VP1	O	Sequencing

Oligonucleotide	Sequence (5'→3')	Sense	Location	Serotype	Method used
O-1D628R	GTTGGGTTGGTGGTGGTTGT	–	VP1	O	Sequencing
O-1D628aR	GTTGGATTAGTGGTGTTAT	–	VP1	O	Sequencing
A-1C562F	TACCAAATTACACACGGGAA	+	VP3	A	RT-PCR
A-1C612F	TAGCGCCGGCAAAGACTTTGA	+	VP3	A	RT-PCR and sequencing
A-1D523R	CGTTTCATRCGCACRAGRA	–	VP1	A	Sequencing
A-1D478bR	AATTGCACCGTAATTGAAGGATGC	–	VP1	A	Sequencing
C-1C536F	TACAGGGATGGGTCTGTGTGTACC	+	VP3	C	RT-PCR
C-1C616F	AAAGACTTTGAGCTCCGGCTACC	+	VP3	C	RT-PCR and sequencing
C-1D535R	ARAGYTCIGCICGYTTCAT	–	VP1	C	Sequencing
SAT 2B208R	ACAGCGGCCATGCACGACAG	-	2B	SAT 1, SAT 2	RT-PCR
SAT1-1C559F	GTGTATCAGATCACAGACACACA	+	VP3	SAT 1	RT-PCR and sequencing
SAT1U-OS	GTGTACCAGATCACTGACAC	+	VP3	SAT 1	RT-PCR and sequencing
SAT1-1D200F	TGCGYGCIGCCACGTACTAYTTCTC	+	VP1	SAT 1	Sequencing
SAT1-1D394R	GGYTTGTACTTRCARTCACCGTTGTA	–	VP1	SAT 1	Sequencing
SAT2-1C445F	TGGGACACMGGIYTGAACTC	+	VP3	SAT 2	RT-PCR
SAT2-P1-1223F	TGAACTACCACTTCATGTACACAG	+	VP3	SAT 2	RT-PCR
SAT2-D	GGTGCGCCGTTGGGTTGCCA	–	VP1	SAT 2	Sequencing
SAT2-1D209cF	CCACCTACTATTTCTGTGACCTGGA	+	VP1	SAT 2	Sequencing
SAT2VP3-AB	CACTGCTACCACTCRGAGTG	+	VP3	SAT 2	Sequencing

*RT-PCR, reverse transcription–PCR; VP virus protein; SAT, Southern African Territories.
